# Potential roles and prognostic significance of exosomes in cancer drug resistance

**DOI:** 10.1186/s13578-020-00515-y

**Published:** 2021-01-06

**Authors:** Mostafa Mostafazadeh, Nasser Samadi, Houman Kahroba, Behzad Baradaran, Sanya Haiaty, Mohammad Nouri

**Affiliations:** 1 Drug Applied Research Center, Tabriz University of Medical Sciences, Tabriz, Iran; 2 Department of Biochemistry and Clinical Laboratories, Faculty of Medicine, Tabriz University of Medical Sciences, Tabriz, Iran; 3 Biotechnology Research Center, Tabriz University of Medical Sciences, Tabriz, Iran; 4 Department of Molecular Medicine, Faculty of Advanced Medical Sciences, Tabriz University of Medical Sciences, Tabriz, Iran; 5 Immunology Research Center, Faculty of Medicine, Tabriz University of Medical Sciences, Tabriz, Iran; 6 Infectious and Tropical Diseases Research Center, Tabriz University of Medical Sciences, Tabriz, Iran; 7 Stem Cell and Regenerative Medicine Institute, Tabriz University of Medical Sciences, Tabriz, Iran

**Keywords:** Biomarker, Chemoresistance, Extracellular vesicles, MicroRNAs, Tumor microenvironment

## Abstract

Drug resistance is a major impediment in cancer therapy which strongly reduces the efficiency of anti-cancer drugs. Exosomes are extracellular vesicles with cup or spherical shape with a size range of 40–150 nm released by eukaryotic cells that contain genetic materials, proteins, and lipids which mediate a specific cell-to-cell communication. The potential roles of exosomes in intrinsic and acquired drug resistance have been reported in several studies. Furthermore, a line of evidence suggested that the content of exosomes released from tumor cells in biological samples may be associated with the clinical outcomes of cancer patients. In this review, we highlighted the recent studies regarding the potential roles of exosomes in tumor initiation, progression, and chemoresistance. This study suggests the possible role of exosomes for drug delivery and their contents in prognosis and resistance to chemotherapy in cancer patients.

## Introduction

Cancer is a major public health problem and the second most common cause of death in the world [1]. Among several therapeutic strategies, chemotherapy is one of the main approaches for tumor treatment [2]. Although significant advances have been developed for increasing the efficacy of chemotherapeutics, chemoresistance remains a major obstacle against the effective treatment of cancer patients [3]. Exosomes are multi-signal messengers that support cancer development, progression, and chemoresistance by mediating the tumor–tumor and tumor–stromal cells interaction [4]. Furthermore, accumulating data suggests that the differential content of exosomes in body fluids can be used as a prognostic factor for cancer therapy and clinical outcomes [5–8]. Here, we describe the structure biological functions of exosomes and their application as nano-carriers for drug delivery. We also discussed how to target these vesicles to increase the effectiveness of chemotherapeutic agents through overcoming chemoresistance.

## Structure, content, and biological roles of exosomes

**Fig. 1:**
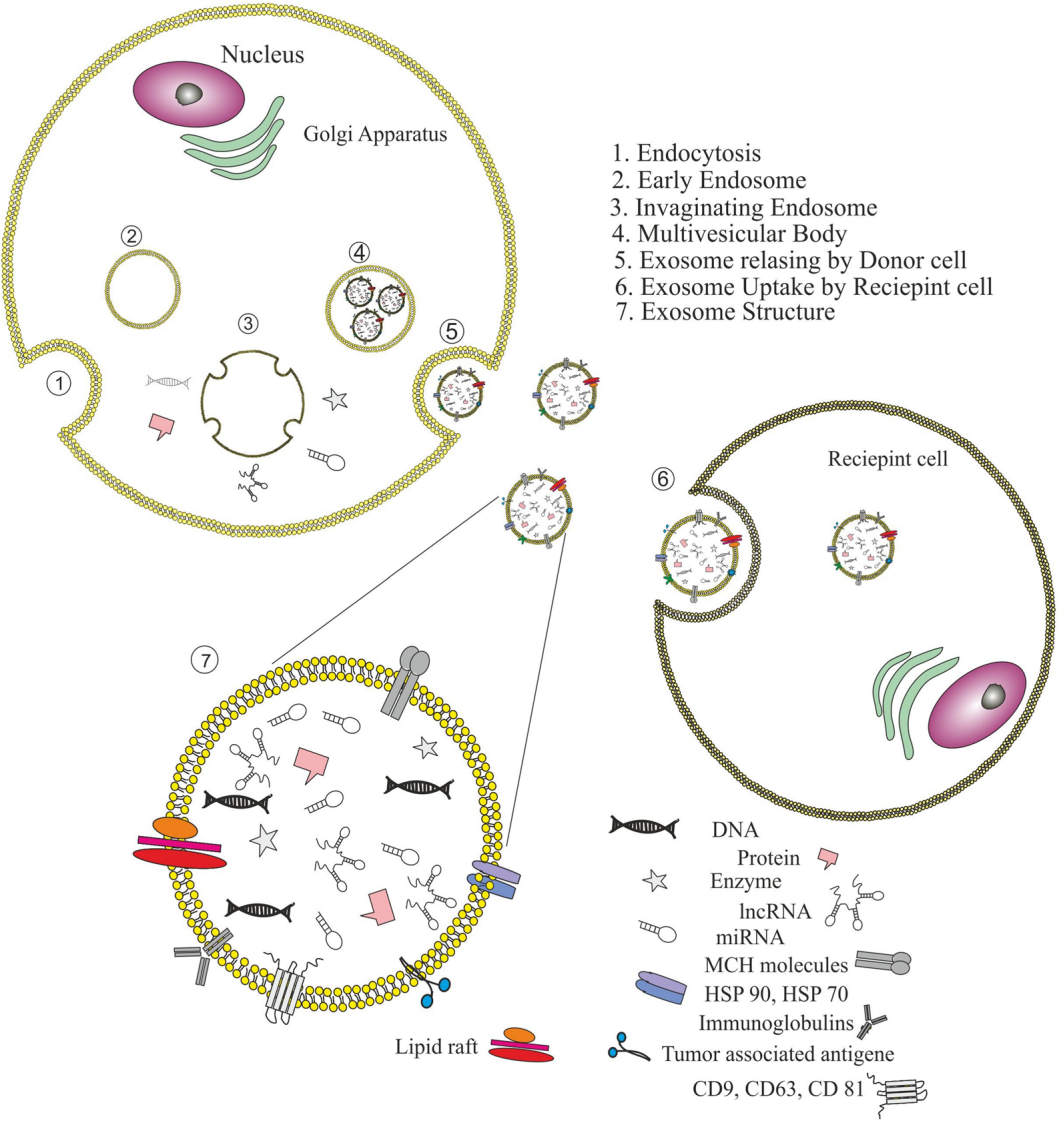
**a** Exosomes are cup/spherical-shaped vesicles (40–150 nm) with a double-layered lipid membrane surrounding a small cytosol without any organelles. This phospholipid bilayer membrane loaded with peripheral and integral proteins. Exosomes also contain nucleic acids (e.g. DNA, mRNAs, microRNAs, and long non-coding RNAs) and lipids. **b** Exosome biogenesis started via endocytosis pathway and early endosome formation. During this process, the cell membrane components (proteins and etc.) are embedded into the early endosomes’ membrane, which then matured into late endosomes. Inward budding of late endosomal membrane creates multiple intraluminal vesicles (ILVs) within multivesicular endosomes, in which some particular proteins and other cytosolic constituents are enveloped in exosomes under control of ESCRT family. MVBs then fuse with the cell membrane to release their ILVs into extracellular milieu by Rab GTPases 27A and B molecular motors

Exosomes are cup-shaped or spherical extracellular vesicles, these nano-sized vesicles span 40–150 nm in diameter and weight 1.13–1.19 g/mL in density. The exosomes are consisting of a double-layered lipid membrane which surrounds a small fraction of cytosolic content but do not include any cytoplasmic organelles. The content of the exosomes is in direct relation to the physiological status of the mother cell [9]. Exosome membrane is enriched with peripheral and integral proteins including multi-vesicular body biogenesis associated proteins (tumor susceptibility gene 101 protein (TSG101), Alix), adhesion molecules (ICAM-1), MHC I and II molecules, GTPases, heat shock proteins (Hsp60, Hsp70, and Hsp 90), Rab proteins, clathrin, tubulin, annexins, flotillin-1, cholesterol, ceramides, sphingomyelin and phosphatidylethanolamine which are crucial for the function of exosome during inter-and intra-cellular communication [10, 11]. Tetraspanins (CD9, CD63, and CD81) are the most common surface markers of the exosomes which are widely applied for exosome detection [12, 13]. these vesicles carry out different macromolecules as their cargo, including DNA, mRNA, microRNA, long non-coding RNA (lncRNAs), lipids, and proteins [10]. Exosome biogenesis initiates during inward budding of late endosomal membrane which results in the formation of intraluminal vesicles (ILVs) within multivesicular endosomes or multivesicular bodies (MVBs). Endosomal sorting complex required for transport proteins (ESCRT-0, I, II, and III) participate in direction of ILVs toward selective cargo loading [14]. Capable MVBs for releasing as exosome can move toward cell membrane and secret ILVs into extracellular milieu under control of Rab GTPases 27A and B molecular motors, also the MVBs which are not capable of releasing as exosome can fuse with lysosomal compartment for degradation and recycling of the components [15]. The general structure, composition, and biogenesis of exosomes are summarized in Fig. 1.

Exosomes can be secreted by almost all eukaryotic cell types, and they are traceable in body fluids such as blood, urine, saliva, cerebrospinal fluid (CSF), amniotic fluid, breast milk, synovial fluid, and ascites [16, 17]. The biological functions of exosomes rely on the cells of origin and the status of the originated tissue or cell at the time of exosome biogenesis. Normally, cells expel toxic or redundant nonfunctional cellular components including drugs via exosomes to maintain the cellular homeostasis [18]. The persistent presence of exosomes in biological body fluids and extracellular spaces suggests their important role in the cell–cell communication network and it has been shown that these nano-vesicles play significant roles in biological processes (e.g. antigen presentation, coagulation, cellular homeostasis, angiogenesis, apoptosis, and synaptic physiology) and various pathological conditions including autoimmune and neurodegenerative diseases, infectious diseases, inflammation, and cancers [16, 19, 20].

## Exosome and cancer drug resistance

**Fig. 2:**
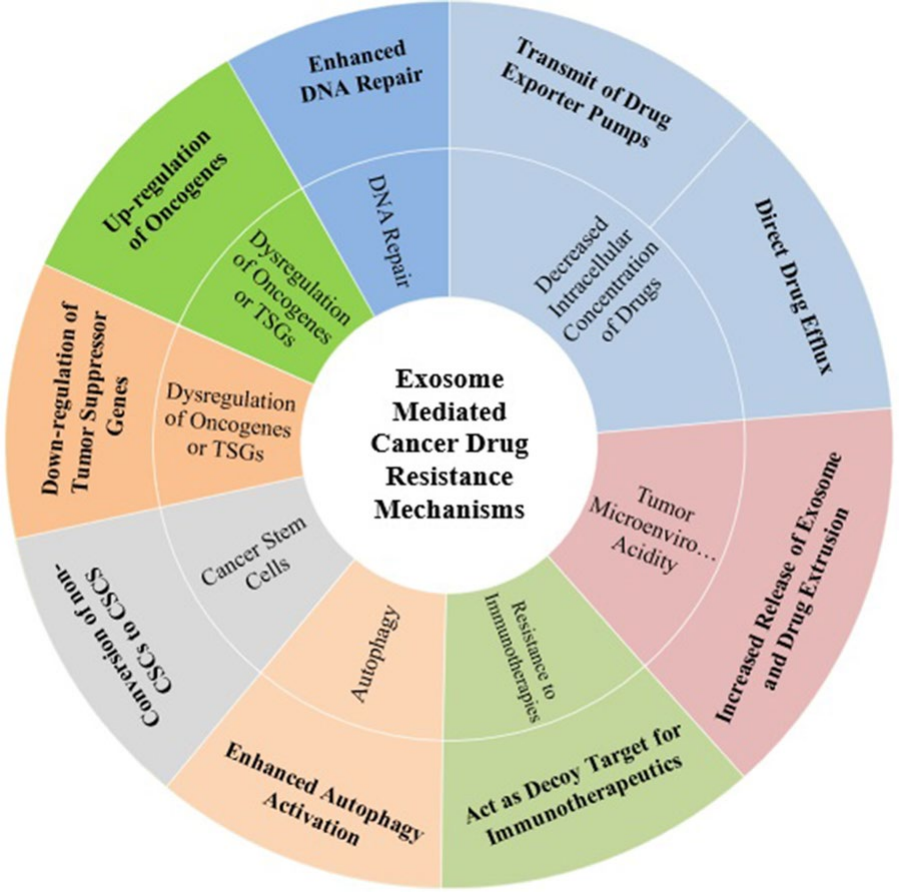
Exosome mediated cancer drug resistance mechanisms

Drug resistance is defined as the reduction in effectiveness and potency of medication to produce therapeutic merits which is a major obstacle in cancer treatment [21]. To the best of our knowledge, resistance to anti-cancer drugs can be categorized into two main classes, intrinsic (pre-existent) and acquired drug resistance [22]. In the intrinsic drug resistance, resistance-associated factors (e.g. presence of cancer stem cells) exist in the tumor mass before any drug exposure [23], while acquired drug resistance or multi-drug resistance (MDR) is a slow and stepwise process which force tumor cells to undergo genetic mutations or epigenetic changes during treatment which results in drug-resistant phenotype [24]. Acquired drug resistance can be attributed to the decreased intracellular concentration of chemotherapeutic agents, altered expression of oncogenes or tumor suppressor genes, enhanced DNA damage repair, epithelial-mesenchymal transition (EMT), autophagy [25, 26], and highly acidic microenvironment of tumors [27]. Recently an ever-increasing body of evidence highlighted the significant role of exosomes in modulating the tumor-specific chemoresistance strategies that lead to the induction of tumor drug resistance [28, 29]. Here, we describe the potential roles of exosomes in the establishment of therapeutic resistance in cancer (Fig. 2).

## Exosomes and drug efflux

Increased efflux of chemotherapeutic agents, which leads to declined intracellular drug concentration, has been considered to be the main cause of drug resistance in cancer [30]. One of the mechanisms by which tumor cells reduce intracellular levels of cytotoxic substances is the secretion of the exosomes to eliminate the cytotoxic effects of anti-cancer agents. Cancer cells can simply prevent the function of these agents and their metabolites by encapsulating them in exosomes to remove out them from the cell [31]. In prostate cancer, Enzalutamide (ENZ)-resistant cells release significantly higher amounts (2–3 fold) of exosomes compared to respective sensitive cells, also it has been shown that these resistant cells use exosomes to remove Enz out of the cell to reduce the drug concentration [32]. Similarly, Koch et al. showed that B-cell lymphoma cells could eliminate doxorubicin and pixantrone through exosome secretion, and that exosome biogenesis inhibition via indomethacin or genetic depletion of ABCA3 enhances intracellular accumulation and cytostatic activity of both drugs in vitro as well as in vivo experiments [33]. Furthermore, Wang et al. indicated that treatment of breast and ovarian cancer cell lines with paclitaxel or doxorubicin significantly increase exosome release in a time and dose-dependent manner, the exosomes isolated from these cells contain a considerable concentration of the drug, and these exosomes have cytotoxic effects on recipient cells [34]. In addition to facilitating drug efflux by exosomes, drug exporter pumps, such as P-glycoprotein (P-gp/MDR1), multidrug-resistant protein-1 (MRP-1), and breast cancer resistance protein (BCRP/ABCG2) participate in drug efflux from the cells, therefore overexpression of these pumps in tumor cells can decrease the drug bioavailability and establish drug resistance [35, 36]. Several studies have shown that exosomal transportation of P-gp induces chemoresistance phenotype in recipient tumor cells [37–44]. For instance, Corcoran et al. revealed that exosomes derived from docetaxel-resistant prostate tumor cells induce docetaxel-resistance in drug-sensitive cells, which may be partially due to exosomal delivery of MDR-1/P-gp. Also, exosomes isolated from the serum of patients with increasing PSA levels (non-respondent to docetaxel) could protect prostate tumor cells from the cytotoxic effects of docetaxel [38]. Another study has shown that exosomes released from doxorubicin-resistant osteosarcoma cells induce a resistant phenotype in recipient cells by delivering MDR-1 mRNA and its product P-glycoprotein [43].

## Exosomes and cancer stem cell-mediated drug resistance

Cancer stem cells (CSCs), a small subset of cancer cells with self-renewal and multi-differentiation capacities, play a critical role in tumor initiation and progression. Recent studies revealed that the presence of CSCs within tumor mass is the main cause of cancer therapy resistance, leading to tumor relapse and ultimately metastasis [45, 46]. Due to their upregulated expression of drug efflux transporters (i.e., P-gp, ABCG2) and other chemoresistance related genes, these highly tumorigenic stem cells are inherently resistant to chemotherapeutic agents [47]. In addition to drug resistance, this rare population has a high tumorigenicity capacity, which enables them to repopulate a tumor after chemotherapy. In this regard, several studies have shown that recurrent tumors after chemotherapy failure are often enriched in cells that expressing CSC markers [48, 49], however, the origins of CSCs are not yet clear and controversial [46]. The accumulated knowledge suggest that CSCs could be derived from cancer cells/differentiated cells that acquired stem-like characteristics through reprogramming and dedifferentiation [50].

A body of evidence confirms that exosomes, as a mediator of intercellular communication in the TME, participate in the conversion of non-CSC to CSCs via delivering stemness and EMT promoting factors. Snail1, a zinc finger transcriptional factor that promotes the repression of E-cadherin expression, is shared by cancer-associated fibroblasts (CAFs) derived exosomes to induce EMT and establishment of lung cancer cells with CSC characteristics [51]. Fibroblasts-derived exosomes contain IL-6, Activin-A, and granulocyte colony-stimulating factor (G-CSF) which are interacted with lung carcinoma cells to induce gene expression alterations, most probably through STAT3 and Smad activation. The consequent activation of stemness-associated pathways such as Wnt, Notch, and Hedgehog direct tumor cells’ dedifferentiation of lung cancer cells to a more CSC-like phenotype and reduces cell cycle progression, which is associated with higher methotrexate resistance [52]. Similarly, exosomal Wnt derived from fibroblasts induces resistance to 5-fluorouracil (5-Fu) via reprogramming differentiated colorectal cancer (CRC) cells to functional CSCs [53].

In addition to exosomal protein cargo, several exosomal miRNAs are also reported to be involved in the EMT–associated chemoresistance. For instance, exosomal delivery of oncomiRNA-155 from CSCs and resistant breast cancer cells to sensitive breast cancer cells mediates the loss of C/EBP-β, which in turn, causes loss of TGF-β and leads to EMT and chemoresistance in recipient cells [54]. CAFs secreted exosomes contain miR-92a-3p which can induce 5-FU/L-OHP resistance by promoting cell stemness and EMT in CRC through targeting FBXW7 and MOAP1 [55]. In CRC, it has been shown that fibroblast-derived exosomes can develop chemoresistance to 5-FU or oxaliplatin through increasing CSCs percentage, clonogenicity, and tumoral growth [29]. Together, these results depict that exosomes could promote the dedifferentiation of cancer cells to CSCs and the development of chemoresistance, and propose that interfering with exosome release and uptake may help to increase chemosensitivity of cancers to available chemotherapeutics.

## Exosomes and autophagy induced drug resistance

Autophagy (or macro-autophagy) is a highly conserved catabolic mechanism for the destruction and recycling of redundant or dysfunctional cellular components, in which a specialized double-layered membrane vesicle called autophagosome is formed around the unnecessary cellular constituent or intracellular pathogen, which eventually fuses with the lysosome to degrade the confiscated material [47]. Autophagy activation normally occurs in cells in response to environmental stressors, facilitates cell survival, and can establish drug resistance in malignant cells [56]. Increased activity of autophagy and improved secretion of exosomes from tumor cells after chemotherapy have been documented in several studies, indicating that these responses are part of the cells’ response to stress conditions due to chemotherapy and survival mechanisms against chemotherapies [57, 58]. In support of this possibility, the potential role of exosomes in autophagy-mediated therapy resistance has been reported in different types of cancers. For example, Gefitinib, a tyrosine kinase inhibitor, is widely used for the treatment of non-small cell lung cancer (NSCLC) patients with EGFR mutations, but its combination with Platinum-based antineoplastic drugs leads to an antagonistic effect. Exosome isolated from gefitinib-treated EGFR-mutant NSCLC cells conditioned medium decreased the anti-neoplastic effects of cisplatin by increasing autophagic activity and reducing apoptosis, as confirmed by an increasing Bcl-2/Bax ratio, upregulation of LC3-II, and downregulation of p62 protein levels [59]. Similarly, exosomal mediated transmit of miR-425-3p enhanced autophagy activation in the recipient NSCLC cells via targeting AKT1, eventually leading to resistance to cisplatin [60]. In hepatic cancers, exosomes secreted from HBV–infected cancer cells can downregulate cell apoptosis and promote oxaliplatin resistance via activating the chaperone-mediated autophagy (CMA) pathway through upregulation of lysosome-associated membrane protein (Lamp2a) as a key molecule in CMA pathway [61]. More interestingly, exosomal transmit of miR-567 from normal breast epithelial cells (MCF-10A) resensitized resistant breast cancer cells to trastuzumab by targeting autophagy-related 5 (ATG5) and thereby inhibiting autophagy [62]. These studies demonstrate the possible capacity of the crosstalk between cancer cells derived exosomes and autophagy phenomenon in cancer therapy response.

## Role of exosomes in dysregulation of oncogenes or tumor suppressor genes expression

Oncogenes and tumor-suppressor genes (TSGs) are two broad classes of genes that play crucial roles in oncogenesis by opposite mechanisms. Oncogenes encoded proteins (Onco-proteins) promote normal cell growth while TSGs encode proteins inhibit the survival of malignant cells. Several reports indicated that TSGs and oncogenes are important mediators of drug resistance [63]. Exosomes have been shown to alter the expression of TSGs and oncogenes through the intercellular translocation of noncoding RNAs.

TP53 is the most studied TSGs and is mutated in more than half of human cancers which participates in cell cycle arrest and apoptosis following DNA damage through transcriptional activation of pro-apoptotic genes or sequestration of anti-apoptotic proteins. Alteration in TP53 expression or its function is often correlated with resistance to standard antineoplastic agents [64]. In prostate cancer, exosomal miR-27a could induce resistance to cisplatin, docetaxel, and doxorubicin in recipient cells by the degradation of p53 mRNA [65]. Down-regulation of PTEN expression, a negative regulator PI3k/Akt signaling pathway, results in reduced dephosphorylation of PIP3 and enhances cell survival and proliferation [63]. Recently, it has been reported that transferring exosomal miR-32-5p from resistant cells to sensitive cells can activate the PI3K/Akt pathway by targeting PTEN and disseminating resistance to 5-FU by promoting angiogenesis and EMT [66]. CAV1 encodes Caveolin1, a negative regulator of EGFR activation, which acts as a tumor suppressor gene in glioblastoma (GBM) cells. Exosomal delivery of bioactive miR-1238 from temozolomide (TMZ) resistant cells can provoke chemoresistance through triggering the EGFR-PI3K-AKT-mTOR pathway in sensitive GBM cells [67]. CAFs are inherently resistant to gemcitabine (GEM) and CAF derived exosomes contain miR-106b which promotes GEM resistance in pancreatic cancer cells by direct targeting of TP53INP1 [68]. Furthermore, exosomal miR-155-5p increases paclitaxel resistance in recipient gastric cancer cells via GATA3 and TP53INP1 downregulation [69].

In addition to miRNAs, tumor-derived exosomes also induce chemoresistance in drug-sensitive cells via transferring of lncRNAs [70, 71]. Sunitinib, a receptor tyrosine kinase (RTK) inhibitor, is the first-line treatment for renal cell carcinoma (RCC) patients, which has potent anti-angiogenic and anti-tumor activities by inhibition of VEGF receptor, PDGF receptor, FMS-like tyrosine kinase 3 (FLT3), and stem cell growth factor receptor. Qu et al. showed that exosomes can share lncARSR which induces sunitinib resistance via competitively binding miR-449/miR-34 to increase c-MET and AXL expression in RCC cells [72]. Similarly, exosomal delivery of lncRNA PART1 disseminates gefitinib resistance in esophageal squamous cell carcinoma by regulating miR-129/Bcl-2 pathway [70]. Treatment of drug-sensitive breast cancer cells with exosomes derived from trastuzumab-resistant cells that highly expressing lncRNA-SNHG14 can confer trastuzumab resistance by targeting the Bcl-2/Bax signaling pathway [73]. Exosomal lncRNA CCAL from CAFs directly interacts with human antigen R (mRNA stabilizing protein HuR), enhances β-catenin expression at both mRNA and protein levels, and promotes oxaliplatin resistance in CRC cells [74].

Besides, exosomes have been shown to directly transduce oncogenes into the sensitive cells to induce drug resistance. For instance, MET is shared by GBM cells harboring PTPRZ1–MET fusion to establish temozolomide resistance in GBM cells [75]. Survivin, a member of the inhibitor of apoptosis (IAP) protein family, can be regarded as an oncogene, aberrant overexpression of this molecule induces resistance to apoptotic stimuli and chemotherapeutic agents. It has been shown that exosomal transferring of survivin promotes paclitaxel resistance in breast cancer cells [76]. Anaplastic lymphoma kinase (ALK) is an RTK that can be activated by mutations and acts as an oncogene in different cancers. Cesi et al. showed that a novel truncated form of ALK can be shuttled by vemurafenib resistant melanoma cell-derived exosomes and promote resistance phenotype in recipient cells [77]. Moreover, exosome-mediated transfer of p-STAT3 from resistant cells significantly promoted cell survival and 5-FU resistance both in vitro and in vivo [78].

## Exosomes and enhanced DNA repair in tumor cells

Most chemotherapeutic agents, including platinum-based drugs, 5-FU, and TMZ, target tumor cells by inducing DNA damage, which can initiate a variety of signaling pathways called DNA damage response (DDR) [79] comprising DNA lesion detection, signal transduction, cell cycle checkpoints activation, and DNA repair, which may elicit resistance to clinical DNA-damaging agents through increasing DNA repair [80]. Also, the potential role of exosomes in enhancing DNA repair and promoting cancer cell survival has been confirmed in several studies. For example, X-ray repair cross complementing 4 (XRCC4), a major factor for double-strand breaks (DSBs) repair, has recently been shown to be connected with TMZ resistance in tumor cells. Zhang et al. revealed that exosomal lncRNA SBF2-AS1 provokes TMZ resistance in recipient cells. Mechanistically, lncRNA SBF2-AS1 acting as a competing endogenous RNA (ceRNA) for miR-151a-3p, leads to the disinhibition of its endogenous target, XRCC4, which enhances DNA lesions repair in tumor cells [81]. In another study, it has been shown that exosome-mediated transferring of miR-151a re-sensitize GBM-resistant cells to TMZ by targeting XRCC4 [82]. MGMT (O^6^-methylguanine DNA methyltransferase) is the key enzyme involved in TMZ-induced DNA damage repairing and its tissue-level expression is negatively correlated with the treatment efficacy. Exosomal delivery of MGMT mRNA from reactive astrocyte to glioma cells confers TMZ resistance phenotype [83].

## Tumor microenvironmental acidity and exosome-mediated chemoresistance

The tumor microenvironment (TME) is acidic, in vivo ranges from 6.5 to 7.1, which is a common phenotype of virtually all tumors and the TME acidity is directly related to the tumor malignancy [27]. Altered glycolytic activity due to the overexpression of M2-PK (a dimeric isoenzyme of pyruvate kinase) and hypoxia, the so-called “Warburg Effect”, causes a large accumulation of lactic acid and proton (H^+^) within the tumor cell cytoplasm, which contributes to TME acidification [84]. Also, high amounts of carbon dioxide are produced during mitochondrial respiration in oxygenated tumor cells, which also contributes to the significant release of protons into the TME [85].

Decreased extracellular pH induces a selective pressure result in the selection of tumor cells that promotes cancer drug resistance [86], which one of the common phenomena associated with this selection is increased exosome secretion [87].

There is strong evidence that a low pH potentially influencing exosome release and uptake by tumor cells [88]. Recently it has been shown that tumor acidity increases exosome secretion in human tumor cells from different cancer, including melanoma, osteosarcoma, colon, prostate, and breast cancer [89]. The underlying mechanism of increased exosome extrusion under acidic conditions is unclear; however, one of the main and well-known functions of the exosome is the removal of toxins, including chemotherapeutics, from the cell cytoplasm [84]. High acidity extracellular environment may likely induce an amplified exosome secretion for detoxifying purposes. In this regard, several investigations have indicated that resistant cancer cells secrete larger amounts of exosomes than their sensitive counterparts [90, 91]. Furthermore, the results of some recent experiments in vitro revealed that the acidic extracellular environment increases the number of released exosomes along with upregulation of certain tumor biomarkers such as carbonic anhydrase IX (CA IX) and prostate specific antigen (PSA) in cancer cells and their exosomes [17, 92].

Therefore, considering the effect of environmental acidity on exosome secretion and chemoresistance, an anti-acid approach can be proposed as a winning strategy in cancer treatment, which may be achieved with buffers or proton pump inhibitors (PPIs) [89]. Recent studies have verified that the alkalization of tumor cell environment with buffers causes a sharp decrease in exosome secretion [93]. Furthermore, two different studies have demonstrated that buffering the tumor environment decelerates tumor growth of xenografts in mice and enhances responsiveness to chemotherapeutics in human and veterinary patients [94, 95]. Moreover, pre-treatment of human melanoma cells Lansoprazole (a PPIs) prevents tumoral exosome secretion leading to improved cisplatin-dependent cytotoxicity [96].

## Exosomes and resistance to immunotherapies

Several studies have suggested that tumor cells produce and secrete exosomes that act as a decoy target for anti-cancer immunotherapies. Trastuzumab (Herceptin), a humanized monoclonal antibody targets the extracellular domain of human epidermal growth factor receptor 2 (HER2) inhibits tumor cells’ survival and proliferation, and is widely used as an approved medication for early HER2 positive and advanced metastatic cancers. It has been shown that HER2-overexpressing breast cancer cells, release HER2-containing exosomes which interfere with the therapeutic activity of Herceptin. Exosomal surface expression of HER2 allows the exosome to compete with breast cancer cells for Trastuzumab binding, thus restraining bioavailability and decreasing Trastuzumab anti-cancer efficiency [97]. In breast cancer, Han et al. showed that exosomal lncRNA AFAP1-AS1 (actin filament associated protein 1 antisense RNA 1) could promote Herceptin resistance through associating AU-binding factor 1 (AUF1) and HER-2 protein levels upregulation [62].

Rituximab, a chimeric antibody against cell surface CD20 antigen, exerts its cytolytic effects via direct induction of apoptosis, antibody-dependent cytolysis, as well as complement-dependent cellular cytotoxicity, and is used in aggressive B-cell lymphoma immunotherapy. B-cell lymphoma cells secrete CD20 carrying exosomes, which bind and deplete therapeutic anti-CD20 antibodies, leads to complement components depletion, and shield cancer cells from antibody attack [98].

## How to combat exosome-induced drug resistance

Given the above, the potential role of exosomes in cancer therapy resistance can be understood, therefore, to maximize the efficacy of chemotherapy, eliminating the detrimental effect of exosomes on cancer therapy seems to be necessary. To this end, several approaches have been proposed including inhibition of exosome biogenesis and release, inhibition of their uptake by recipient cells, and targeting their cargo. Some pharmacological and chemical compounds have been used to sensitize cancer cells to chemotherapeutic agents via targeting biogenesis, release, and exosome uptake which have been summarized in Table 1. For instance, in leukemia and lymphoma, it has been found that high expression of ATP-binding cassette transporter A3 (ABCA3) is crucial for exosome generation and chemoresistance and pretreatment of diffuse large B-cell lymphomas (DLBCL) with indomethacin, a COX inhibitor, enhances the accumulation of doxorubicin and pixantrone in the nuclei of DLBCL tumor cell through the reduction in ABCA3 levels and exosome biogenesis, which increases tumor cell susceptibility to these drugs [33]. Manumycin A, a natural microbial metabolite with a potent selective Ras farnesyl-transferase inhibitory activity, decrease exosome biogenesis and secretion by inhibition of Ras/Raf/ERK1/2 signaling pathway and consequent inhibition of the oncogenic splicing factor hnRNP H1 in castration-resistant prostate cancer cells [99]. In another study, it has been shown that pre-treatment of tumor cells (MCF7, HeLa, and BT549) with ketotifen, an anti-histamine which acts as a calcium channel blocking agent, reduces exosome release and increases the sensitivity of cancer cells to doxorubicin by enhancing intracellular drug retention [100]. Similarly, 5-FU mediated apoptosis was significantly increased in prostate and breast cancer cell lines in the presence of a combination of chloramidine and bisindolylmaleimide-I, which act as inhibitors of exosome release [101]. Exosomes interact and are taken up by recipient cells in various mechanisms such as direct integration with the cell membrane, clathrin and caveolin1-mediated endocytic pathways, cholesterol/lipid rafts–dependent endocytosis, macro-pinocytosis, phagocytosis, and ligand-receptor interaction. As a result, targeting the aforementioned mechanisms can prevent the internalization of the exosomes. On such a basis, several researchers have attempted to overcome the chemotherapy resistance through inhibition of exosome uptake by target cells. To et al. reported that exosome uptake inhibition by dynasore, dynamin GTPase activity inhibitor, prevents ABCG2 induction and sensitizes the resistant colorectal cancer cells to SN38 treatment [102]. Heparan sulfate proteoglycans (HSPGs) acts as cell surface receptors for exosome uptake and internalization [103]. Pre-treatment of oral squamous carcinoma cells (OSCCs) with heparin, a competitive inhibitor of HSPG mediated endocytosis, inhibits exosome uptake by recipient cells and suppresses the exosome induced tumor progression in vitro and in vivo [104].

**Table 1:** Chemical and pharmacological agents for targeting exosome biogenesis, secretion, and uptake

	Drug/Compound	Mechanism	Ref.
Inhibition of exosome biogenesis and secretion	GW4869	Targeting neutral sphingomyelinase 2	[29]
	Manumycin	Inhibition of Ras/Raf/MEK/ERK1/2 signaling pathway	[99]
	Indomethacin	Blocking the expression of ABCA-3	[33]
	Ketotifen	Unknown	[100]
	Chloramidine and bisindolylmaleimide-I	Unknown	[101]
	Lansoprazole (proton pump inhibitor)	Decreasing microenvironmental acidity	[96]
Inhibition of exosome internalization	Heparin	Inhibition of HSPG mediated endocytosis	[104]
	Cytochalasin D	Inhibition of actin polymerization	[105]
	Dynasore	Inhibition of the GTPase activity of dynamin	[106]
	Chlorpromazine	Inhibition of clathrin-dependent endocytosis	[107]
	Methyl-β-cyclodextrin	Remove cholesterol from the cell membrane	[108]
	Nystatin and Simvastatin*	Inhibition of the lipid raft -mediated endocytosis pathway/*Inhibitor of HMG-CoA	[109]
	Filipin III	Inhibitor of lipid raft dependent and caveolar endocytosis	[88]

In addition to inhibiting biogenesis, release, and uptake of exosomes, exosome cargo targeting could also be an approach to overcome exosome-induced drug resistance. In esophageal cancer, exosome-mediated transferring of MiR-21 provokes cisplatin-resistant phenotype in recipient cells through targeting programmed cell death 4 (PDCD4) mRNA and downregulating its protein level. It has been shown that cell transfection with anti-miR-21 (the antisense oligonucleotide sequence of miR21) inhibits exosome induced chemoresistance, which raises the possibility that anti-exosome miR-21 can act as a potentially useful target for overcoming cisplatin resistance in patients with esophageal cancer [110]. Similarly, in vitro and in vivo inhibition of exosomal miR-214 with antagomir, re-sensitize resistant NSCLC cells to gefitinib [111]. As previously mentioned, the intercellular transmission of lncARSR by exosomes promotes sunitinib resistance in renal cell carcinoma cells, and lncARSR targeting with locked nucleic acids significantly rescinded the sunitinib-exosome-mediated resistance [72].

## Exosomes as a natural capsule for drug delivery to overcome tumor drug resistance

The role of exosomes as a natural vehicle of protein, mRNA, and noncoding RNAs among cells, leads to the idea that they can be used as a delivery system for overcoming chemoresistance. In this context, there is a considerable body of research that shows the efficacy of exosomes in drug or gene delivery due to their ability to pass through the lipid bilayer cell membrane. Exosomes as a natural product of the body have many advantages over the other synthetic nano-carriers for drug or gene delivery, such as low immunogenicity, high biocompatibility and, and high efficacy of delivery [112]. Also, exosomes have good stability in the circulation, which allows them to travel long distances within the body and deliver their cargo to target cells under both physiological and pathological conditions. Furthermore, exosomes have a cytosol like core, which makes them suitable carrier for water-soluble drugs [113]. Today, these natural nanoparticles are widely used as delivery systems for conventional drugs, genes, and other natural compounds. The successful delivery of paclitaxel by exosomes in vitro laid the foundation for exosomes carrying anticancer drugs for in vivo tumor therapy [112]. Kim et al. demonstrated that the incorporation of paclitaxel into exosomes increases cytotoxicity more than 50 times in drug-resistant (P-glycoprotein+) cells [114]. Saari et al. also showed that exosome-mediated delivery of paclitaxel enhances the cytotoxicity of this drug in autologous prostate cancer cells [115]. Several studies have shown that exosomal encapsulation of doxorubicin reduces the doxorubicin-induced cardiotoxicity in mice. Therefore, a higher concentration of doxorubicin can be used for cancer treatment, which in turn increases the efficiency of doxorubicin [116–118]. Moreover, Yang et al. have reported that brain endothelial cell-derived exosomes can pass anticancer agents across the blood-brain barrier (BBB) for the treatment of brain tumors [119]. More interestingly, in another study cow milk-derived exosomes have been used for delivery of the chemo-preventive and chemotherapeutic drugs. In this report, paclitaxel and docetaxel-loaded exosomes showed significantly higher efficacy compared to the free drug in cell culture studies and against lung tumor xenografts in vivo [120]. Acridine Orange (AO) is an organic acidophilic dye with a potent tumor-killing effect after activation by a light source at 466 nm (blue light). However, the clinical use of OA is restricted by its potential systemic toxicity. Iessi et al. demonstrated the exosome-mediated transfer of OA increase the delivery and the efficacy of AO in human melanoma cells [121].

In addition to conventional drugs, various genetic materials such as short interfering RNA (siRNA) and miRNA can also be delivered by exosomes to overcoming drug resistance in tumor cells. Sorafenib is an effective clinical drug in the treatment of hepatocellular carcinoma (HCC) and GRP78 (a member of the HSP family) which is overexpressed in sorafenib resistant cancer cells compared to sensitive cells. Exosomal delivery of siRNA against GRP78 (siGRP78) suppresses sorafenib resistance in HCC [122].

Further studies have indicated that miRNAs can also be delivered by exosomes. For example, in triple-negative breast cancer cells, miR-134-enriched exosomes derived from miR-134-transfected cells decreased aggressiveness, migration, and enhanced sensitivity to anti-Hsp90 drugs in parent cells (with low endogenous miR-134 levels) via down-regulation of STAT5B-Hsp90 [123]. Similarly, Zeng et al. indicated that exosome-mediated transferring of miR-151a to temozolomide-resistant glioblastoma cells enhances chemo-sensitivity to temozolomide [82]. As well as, exosomal miR-122 and miR-199 delivery by mesenchymal stem cell-derived exosomes improved the chemosensitivity of HCC cells respectively to sorafenib [124] and 5-fu [125] in vitro and in vivo studies. It has been shown that miR-9 is involved in the expression of P-glycoprotein and exosomal transferring of anti-miR-9 could confer chemo-sensitivity in glioblastoma multiform cells [126]. Furthermore, treatment of HepG2 cells with miR-744-enriched exosomes inhibited proliferation and sorafenib resistance in HCC through targeting paired box gene 2 (PAX2) [127]. Similarly, exosomes derived from the conditioned media of transfected bone marrow stromal cells with a miR-146b expression plasmid significantly reduces tumor growth in vitro and a rat xenograft model of brain tumor [128]. More excitedly, Liang et al. used engineered exosomes for targeted co-delivery of 5-FU and miR-21 inhibitor to reverse chemoresistance in colon cancer. They showed that exosomal co-delivery of 5-FU and miR-21 inhibitor effectively resensitize resistant cells to 5-FU [129].

## Exosomes as predictive biomarkers for clinical outcome of chemotherapy

Multi-drug resistance, undesirable chemotherapeutic agents’ side effects, and tumor cell dissimilarity among and within the cancer patients restrict the efficacy of anti-cancer drugs. To overcome these obstacles, predictive biomarkers (e.g. tumor-derived exosomes) have recently emerged to guide oncology specialists in the selection of proper chemotherapy drugs for the treatment of various cancer patients [130]. Exosomal cargo properly reflects the features of releasing cells and their metabolic status. For this reason and because of their abundance in biological fluids, accessibility, high circulation stability, selective cargo sorting, and reproducibility, exosomes represent a valuable and non- or semi-invasive biomarker for cancer diagnosis and prognosis [131–134]. There is an increasing interest in using tumor-derived exosomes as easily accessible biomarkers in predicting the clinical outcome of chemotherapy [8, 135–137]. A recently conducted study suggested that high exosomal expression of the oncogenic lncRNA UCA1 (urothelial carcinoma-associated 1) might predict cetuximab resistance in CRC patients [137]. A panel of serum-derived exomiRs (miR-96-5p, miR-21-5p, miR-1229-5p, and miR-1246) is suggested as a predictive biomarker for chemoresistance in CRC patients [138]. Elevated serum-derived exomiR-92a-3p is proposed as a prognostic marker for metastasis and 5-Fu/Oxaliplatin resistance in CRC patients [55]. Modified fluorouracil, leucovorin, and oxaliplatin (mFOLFOX6)-based chemotherapy is the first-line treatment for metastatic CRC. It has been reported that plasma exomiR-125b levels may serve as a useful biomarker for mFOLFOX6)-based first-line chemotherapy resistance in advanced and recurrent CRC cancer patients [139]. Microtubule-targeting chemotherapy agents such as docetaxel and paclitaxel are currently used as the first-line chemotherapy for castration-resistant prostate cancer (CRPC) patients. Serum derived exosomes containing CD44v8-10 mRNA, have been proposed as a potential element for docetaxel-resistance predicting among prostate cancer patients [140]. Circulating exosomal Integrin β4 (ITGB4) and vinculin (VCL) could predict taxane-resistance in prostate cancer patients [141]. Higher plasma levels of exomiRs (miR-1290 and miR-34a) are related to poor response to docetaxel in metastatic CRPC patients [142, 143]. P-gp, which acts as a drug exporter pump, contributes to the expansion of drug resistance in different cancer types. It has been suggested that serum exosomal P-gp in prostate cancer patients could be a useful marker for docetaxel resistance diagnosis [39]. Another study has shown that resistance to hormonal therapy in metastatic prostate cancer patients may be predicted by detecting androgen receptor splice variant 7 (AR-V7) in plasma-derived exosomal RNA [144].

Wang et al. demonstrated that circulating exosome carrying transient receptor potential channel 5 (TRPC5) might act as a noninvasive chemoresistance biomarker for breast cancer patients [145]. Glutathione S-transferase P1 (GSTP1), a xenobiotic-metabolizing enzyme that plays an important role in the detoxification of chemotherapy agents through conjugating them with glutathione, has been shown that enriched in adriamycin-resistant breast cancer cell-derived exosomes and plasma-derived GSTP1-containing exosomes could predict response to chemotherapy drugs in breast cancer patients [136]. Exosomal enrichment of lncRNA-SNHG14 and survivin in human serum is another diagnostic biomarker for drug-resistant breast cancer [73, 76].

In pancreatic cancer, exosomal expression of Ephrin type-A receptor 2 (EphA2) could serve as a minimally-invasive predictive biomarker for responding to GEM [146]. Clinically, high levels of lncSBF2-AS1 in serum exosomes were associated with poor response to TMZ treatment in GBM patients [81]. Different exosomal cargos including miRNAs, LncRNAs, and proteins are previously described as predictive markers in tumor drug resistance which are listed in Table 2.

**Table 2:** Predictive role of exosomal cargo in tumor drug resistance

Cancer type	Exosomal contents (srug)	Ref.
Colorectal cancer	miR-92a-3p (5-Fu/Oxaliplatin)	[55]
	miR–125b (mFOLFOX6)	[139]
	lncRNA UCA1 (Cetuximab)	[137]
	A panel of miR-21-5p, miR-1246, miR1229-5p and miR-96-5p (oxaliplatin and 5-fluorouracil)	[138]
Prostate cancer	ITGB4 and Vinculin (Taxane)	[141]
	AR-V7 (Hormonal Therapy)	[144]
	P-glycoprotein (Docetaxel)	[39]
	CD44v8-10 mRNA (Docetaxel)	[140]
	MDR-1, MDR-3, endophilin-A2, and PABP4 (Docetaxel)	[147]
	miR-1290 (Docetaxel)	[148]
	miR-34a (Docetaxel)	[142]
Breast cancer	Survivin (Paclitaxel)	[76]
	TRPC5	[145]
	TK1 and CDK9 mRNA (CDK4/6 inhibitors)	[149]
	lncRNA–SNHG14 (Trastuzumab)	[73]
	lncRNA AFAP1-AS1 (Trastuzumab)	[62]
	RNA H19 (Doxorubicin)	[150]
	GSTP1 (Adriamycin)	[136]
Multiple myeloma	PSMA3 and lncPSMA3-AS1 (Bortezomib)	[151]
Ovarian cancer	Plasma gelsolin (pGSN)	[152]
Pancreatic cancer	EphA2 (Gemcitabine)	[153]
	miR-155 (Gemcitabine)	[154]
Head and neck cancer	miR-196a (Cisplatin)	[155]
Lung cancer	miR-222-3p (Gemcitabine)	[156]
	miR-146a-5p (Cisplatin)	[157]
	miR-21 (Cisplatin)	[158]
	miR-425-3p (Platinum-based chemotherapy)	[8, 60]
	lncRNA RP11–838N2.4 (Erlotinib)	[159]
Melanoma	PDGFR-B (Vemurafenib)	[160]
Glioblastoma	miR-1238 (Temozolomide)	[161]
	miR-151a (Temozolomide)	[82]
	MET and p-MET (Temozolomide)	[75]
	lncSBF2-AS1 (Temozolomide)	[81]
Gestational trophoblastic neoplasia	miR-219a-5p (Methotrexate)	[162]
Diffuse large B-cell lymphoma	miR-146a	[163]
	miR-99a-5p and miR-125b-5p (R-CHOP regimen)	[164]
Renal cell carcinoma	lncARSR (Sunitinib)	[72]

## Conclusion

The rapid development of drug resistance in tumor cells is one of the most important barriers to cancer treatment, and success in this field depends on a thorough understanding of the molecular mechanisms involved in drug resistance, as well as the complexities of interaction between different components of the tumor microenvironment. Exosomes are intracellular endosomal origin nano-vesicles that are produced and secreted by all eukaryotic cell types in normal and pathological conditions and play a key role in maintaining cellular homeostasis, as well as intra and intercellular communication. Distribution of biological molecules ranging from non-coding RNAs to functional proteins through tumor cellular components by exosomes suggesting their significant role in tumor initiation and chemoresistance expansion. Exosome properties such as presence in all body’s biological fluids, accessibility, high stability, and high sensitivity to reflecting the characteristics of the originating cells, has made exosomes an excellent biomarker for diagnostic and prognostic goals. In this regard, we provide a list of exosomal cargos that can be used as predictor markers of drug resistance. Exosomes as a natural capsule can also be a good tool for drug or gene delivery to overcome tumor drug resistance. Also, exosomes are suitable therapeutic targets for maximizing the efficacy of chemotherapy, therefore, searching for new chemicals or pharmacological agents that interfere with the biogenesis, secretion, and uptake of exosomes by the recipient cells is essential.

## Data Availability

Not applicable.

## Abbreviations

ABCA3: ATP-binding cassette transporter A3
ALK: Anaplastic lymphoma kinase
AR-V7: Androgen receptor splice variant 7
AUF1: AU-binding factor 1
BBB: Blood–brain barrier
BCRP: Breast cancer resistance protein
CAFs: Cancer-associated fibroblasts
ceRNA: Competing endogenous RNA
CSCs: Cancer stem cells
CSF: Cerebrospinal fluid
CRC: Colorectal cancer
CRPC: Castration-resistant prostate cancer
DDR: DNA damage response
DSBs: Double-strand breaks
DLBCL: Diffuse large B-cell lymphomas
EMT: Epithelial-mesenchymal transition
ENZ: Enzalutamide
EphA2: Ephrin type-A receptor 2
ESCRT: Endosomal sorting complex required for transport proteins
FLT3: FMS-like tyrosine kinase 3
5-Fu: 5-Fluorouracil
GBM: Glioblastoma
G-CSF: Granulocyte colony stimulating factor
GEM: Gemcitabine
GSTP1: Glutathione S-transferase P1
HCC: Hepatocellular carcinoma
HER2: Human epidermal growth factor receptor 2
HSPGs: Heparan sulfate proteoglycans
Hsps: Heat shock proteins
IAP: Inhibitor of apoptosis
ILVs: Intraluminal vesicles
ITGB4: Integrin β4
lncRNAs: Long non-coding RNA
MDR: Multi-drug resistance
MGMT: Methyl guanine DNA methyltransferase
MRP-1: Multidrug-resistant protein-1
MVBs: Multivesicular bodies
NSCLC: Non-small cell lung cancer
OSCCs: Oral squamous carcinoma cells
P-gp: P-glycoprotein
PDCD4: Programmed cell death 4
RTK: Receptor tyrosine kinase
siRNA: Short interfering RNA
TMZ: Temozolomide
TSGs: Tumor-suppressor genes
TSG10: Tumor susceptibility gene 101 protein
TRPC5: Transient receptor potential channel 5
VCL: Vinculin
XRCC4: X-ray repair cross complementing
TME: Tumor microenvironment
